# Family medicine graduates' perceptions of intimidation, harassment, and discrimination during residency training

**DOI:** 10.1186/1472-6920-11-88

**Published:** 2011-10-24

**Authors:** Rodney A Crutcher, Olga Szafran, Wayne Woloschuk, Fatima Chatur, Chantal Hansen

**Affiliations:** 1 Department of Family Medicine, University of Calgary, 2500 University Drive NW Calgary, Alberta, T2N 1N4, Canada; 2 Department of Family Medicine, University of Alberta, 901 College Plaza, Edmonton, Alberta, T6G 2C8, Canada; 3 Undergraduate Medical Education, University of Calgary, 2500 University Dr NW Calgary, Alberta, T2N 1N4, Canada

## Abstract

**Background:**

Despite there being considerable literature documenting learner distress and perceptions of mistreatment in medical education settings, these concerns have not been explored in-depth in Canadian family medicine residency programs. The purpose of the study was to examine intimidation, harassment and/or discrimination (IHD) as reported by Alberta family medicine graduates during their two-year residency program.

**Methods:**

A retrospective questionnaire survey was conducted of all (n = 377) family medicine graduates from the University of Alberta and University of Calgary who completed residency training during 2001-2005. The frequency, type, source, and perceived basis of IHD were examined by gender, age, and Canadian vs international medical graduate. Descriptive data analysis (frequency, crosstabs), Chi-square, Fisher's Exact test, analysis of variance, and logistic regression were used as appropriate.

**Results:**

Of 377 graduates, 242 (64.2%) responded to the survey, with 44.7% reporting they had experienced IHD while a resident. The most frequent type of IHD experienced was in the form of inappropriate verbal comments (94.3%), followed by work as punishment (27.6%). The main sources of IHD were specialist physicians (77.1%), hospital nurses (54.3%), specialty residents (45.7%), and patients (35.2%). The primary basis for IHD was perceived to be gender (26.7%), followed by ethnicity (16.2%), and culture (9.5%). A significantly greater proportion of males (38.6%) than females (20.0%) experienced IHD in the form of work as punishment. While a similar proportion of Canadian (46.1%) and international medical graduates (IMGs) (41.0%) experienced IHD, a significantly greater proportion of IMGs perceived ethnicity, culture, or language to be the basis of IHD.

**Conclusions:**

Perceptions of IHD are prevalent among family medicine graduates. Residency programs should explicitly recognize and robustly address all IHD concerns.

## Background

Residency training can be stressful. Some stress is expected and is part of the steep professional and personal learning curve that residents must negotiate prior to commencing independent medical practice. Sometimes stress can arise unexpectedly in the midst of learning and clinical work and can threaten personal and professional wellbeing [1-3]. Stress can take many forms. While some stress can stimulate learning (a student or resident recognizing a knowledge gap at the point of care, and then taking steps to address this), other stress can be potentially impairing (a learner receiving voluminous emotionally charged and non-constructive critique). The shadow side of stress may take the form of intimidation, harassment or discrimination (IHD).

The College of Family Physicians of Canada (CFPC) recognizes the importance of the learning environment to medical education, the stressors embedded in residency training, and considers this "an important and often overlooked component of a training program" [4]. The Standards for Accreditation of Residency Programs of the CFPC state: "a supportive learning environment requires respect for learners, a respect for their learning objectives and a willingness to help them achieve those objectives" [4]. Furthermore, these CFPC standards require "that resources are available to meet the health and safety needs of residents and, in particular, to recognize and provide counselling for stress-related problems among residents" [4].

There is substantial literature documenting the prevalence of IHD in the medical education environment [5-19]. Studies have been conducted on abuse across residency disciplines [20] and sexual harassment within a family medicine residency program [21]; however, concerns surrounding IHD have not yet been explored in-depth in Canadian family medicine residency programs. IHD has been reported by medical students and residents to range between 40-93% in Canada, the USA, the UK, Israel, Japan [8-11,20,22-25]. The most commonly encountered unwanted behaviours were persistent attempts to belittle and undermine the work of the trainee or to humiliate the trainee in front of colleagues [8,26]. Some believe that belittling residents is a "salutary rite of passage" [27] and that intimidation and harassment is potentially an effective educational tool [28].

Experiences of IHD are often not reported usually due to fear of retaliation [23], especially if the perpetrator is also responsible for in-training evaluation. Residents may choose to deal with IHD incidents informally or alone, fearing the consequences of making the offender aware of reported concerns and risking further IHD, if ongoing interaction with that individual is likely. Younger or less mature residents may hold themselves responsible for their own victimization.

Experiences of intimidation and harassment can cause psychological concerns, creating distress and dissatisfaction in the training environment [1,29]. IHD has been found to affect the health of those in training resulting in poor morale and motivation, poor patient care, lower career satisfaction, and mental health problems [1,2,8-10,30,31]. Depressed residents were more than six times as likely to make errors in medication as compared to their non-depressed colleagues [3].

The impetus for our study was threefold: experiential, opportunistic, and quality driven. Firstly, while a family medicine residency program director, one of the authors (RC) became aware of isolated instances of IHD experienced by family medicine residents. This generated two haunting questions: What was actually happening within the program? What would it take to find out? Secondly, the groundbreaking survey work on resident wellbeing by a psychiatry colleague revealed disturbing results regarding resident stress and resident perceptions of harassment and intimidation [1,9]. It was evident that data on IHD among family medicine residents in Canada is scarce and there was no literature comparing IHD between Canadian and IMG family medicine residents. Finally, the pan Canadian work on accreditation and the issue of IHD in postgraduate medical education was being increasingly recognized [32]. Therefore, as part of a sustained and provincial family medicine residency program quality improvement process, it became necessary to formally explore IHD issues in our family medicine settings to provide the evidence-base for any needed action.

The purpose of our study was to examine the frequency, type, source, and basis of intimidation, harassment or discrimination (IHD) during residency training as experienced by Alberta family medicine graduates.

## Methods

### Design & Participants

A retrospective questionnaire survey was conducted of all family medicine graduates from the University of Alberta and University of Calgary who had completed the residency program during 2001-2005, inclusive. The total sample size was 377 graduates for whom contact information was known. A modified Dillman method was used for mailed surveys [33,34]. Each department conducted the mailout to its own graduates. Contact information for the graduates was obtained from the Alberta Medical Directory or the 2006 Canadian Medical Directory.

### Setting

The family medicine residency program at the University of Alberta and University of Calgary is a two-year program. During 2001-2005, the core curriculum consisted of educational experiences (rotations) in family medicine, internal medicine, pediatrics, emergency medicine, coronary care, intensive care, women's health, general surgery, geriatrics, orthopedics, palliative care, psychiatry, and a choice of electives/selectives. In compliance with Canadian family medicine residency program accreditation standards (September 2006) a minimum of eight months of the two-year program was based in family medicine teaching practice settings. Residents were trained in a multidisciplinary health care environment and were in contact with physicians, nurses, various allied health professionals, and patients in both the hospital and community practice settings, and in urban, regional and rural environments.

### Questionnaire

The 2001-2005 thirteen page questionnaire was based on two previous questionnaires used to survey the 1985-1995 and 1996-2000 Alberta family medicine graduates. All three versions of the questionnaire addressed five baseline themes: (a) medical education; (b) career history (clinical and non-clinical activities; current practice, practice location); (c) community involvement; (d) family medicine program evaluation; and (e) demographics. Questions exploring the theme of IHD were added in the 2001-2005 survey. The format and focus of these questions were based on those in the "Happy Doc" study [1,9]. In keeping with "Happy Doc" study protocol and rationale, we chose not to define IHD. The questions addressed the frequency and type/nature of mistreatment, as well as the source and perceived basis of the IHD. Respondents had opportunity to check "other" and make free text comments to specify their beliefs on the source, form and basis of any perceived IHD. Participants were also asked if they were aware of the process to address issues of IHD.

The questionnaire examined IHD as perceived by the respondents and did not distinguish between intimidation, harassment or discrimination. The terms intimidation, harassment, discrimination, and mistreatment were explicitly linked and were used interchangeably in this study. To give definitional context, broadly these terms refer to remarks, actions, or behaviours that are perceived to be unwanted, hurtful, upsetting, or coercive in nature.

The paper version of the questionnaire was pilot-tested on 10 graduates who were not included in the current study sample and refined based on feedback received. A web-based version of the questionnaire was also developed. Both versions of the questionnaire were distributed beginning November 1, 2006 and responses were received until May 31, 2007.

### Data Analysis

SPSS 15 was used for descriptive analysis (frequency, crosstabs), along with Chi-square, Fisher's Exact test, analysis of variance (ANOVA) and logistic regression. An alpha level of 0.05 was used to test for statistical significance. Free text responses were analyzed via a frequency count.

The study was approved by the Health Research Ethics Board (Health Panel) at the University of Alberta and the Conjoint Health Research Ethics Board at the University of Calgary.

## Results

A total of 242 (64.2%) of 377 graduates responded to the survey. The questions on IHD were completed by 235 (97.1%) respondents. The average age of the respondents was 34.6 (SD = 5.3) years, 53.2% were females, and 16.6% were international medical graduates (Table 1).

**Table 1:** Characteristics of Respondents

	Respondents n = 235 (%)		Perceived IHD n = 105 (%)	
Gender*				
Male	109	(46.4)	44	(41.9)
Female	125	(53.2)	60	(57.1)
Not Recorded	1	(0.4)	1	(1.0)
Age**				
≤ 29 yrs	24	(10.2)	13	(12.4)
30 - 34 yrs	124	(52.8)	60	(57.1)
35 - 39 yrs	38	(16.6)	14	(13.3)
40 - 44 yrs	31	(13.2)	14	(13.3)
45 - 49 yrs	9	(3.8)	1	(1.0)
50 - 55 yrs	4	(1.7)	1	(1.0)
Not Recorded	5	(2.1)	2	(1.9)
**Marital Status**^†^				
Single (no children)	44	(18.7)	20	(19.0)
Single (children)	4	(1.7)	1	(1.0)
Married/Common Law (no children)	60	(25.5)	35	(33.3)
Married (children)	125	(53.2)	47	(44.8)
Not Recorded	2	(0.9)	2	(1.9)
**Medical School Graduate**^&^				
Canadian	191	(81.3)	88	(83.8)
International Medical Graduate (IMG)	39	(16.6)	16	(15.2)
Not Recorded	5	(2.1)	1	(1.0)

* p = 0.30 ** p = 0.20 ^† ^p = 0.053 ^&^p = 0.69

All Chi-square analyses with not recorded data excluded in the calculation.

### IHD Prevalence and Frequency

A total of 105 (44.7%) graduates reported that they had experienced IHD while a resident (Table 1); with 36 (34.3%) experiencing IHD only once during residency and 65 (61.9%) experiencing it more than once. Four (3.8%) respondents did not report frequency of IHD.

### Type, Source and Basis of IHD

The most frequent type of IHD experienced by family medicine graduates was in the form of inappropriate verbal comments (94.3%), followed by work as punishment (27.6%) (Table 2). Sexual harassment occurred with the lowest frequency (2.9%). The "other" category (12.4%) included rudeness, hostility, belittling and being excluded from team functions.

**Table 2:** Overall Intimidation, Harassment & Discrimination (IHD)

	Number (%)	
**Type of IHD/n = 105**		
Inappropriate verbal comments	99	(94.3)
Work as punishment	29	(27.6)
Privileges/opportunities taken away	19	(18.1)
Recrimination for reporting	7	(6.7)
Inappropriate/unwanted physical contact	5	(4.8)
Sexual harassment	3	(2.9)
Other	13	(12.4)
**Source of IHD/n = 105**		
Specialist Physicians	81	(77.1)
Hospital Nurses	57	(54.3)
Specialty Residents	48	(45.7)
Patients	37	(35.2)
Family Physicians	19	(18.1)
Support Staff	14	(13.3)
Family Medicine Residents	7	(6.7)
Program Director	4	(3.8)
Family Medicine Nurses	2	(1.9)
**Perceived Basis for IHD/n = 105**		
Gender	28	(26.7)
Ethnicity	17	(16.2)
Culture	10	(9.5)
Language	5	(4.8)
Sexual Orientation	1	(1.0)
Other	80	(76.2)
		

The main sources of IHD were specialist physicians (77.1%), hospital nurses (54.3%), specialty residents (45.7%), and patients (35.2%) (Table 2). Overall, the primary basis for IHD was perceived to be gender (26.7%), followed by ethnicity (16.2%), and culture (9.5%) (Table 2). Many respondents (76.2%) reported "other" reasons that included age, family medicine as a career choice, stress, power hierarchy, a rite of passage, and personality conflict. Just over half (54.4%) of respondents indicated that they were aware of the process to address issues related to IHD within the residency program.

### Gender

There was no significant difference in the prevalence of IHD by gender (males 40.4%, 44/109; females 48.0%, 60/125 (Table 1); however, gender differences were observed for both the type and perceived basis of IHD. A significantly greater proportion of males (38.6%) than females (20.0%) experienced IHD in the form of work as punishment. In contrast, a significantly greater proportion of females (26.7%) than males (6.8%) experienced IHD in the form of privileges/opportunities being taken away. More females (41.7%) than males (4.5%) perceived gender to be the basis for the IHD. Gender differences in source of IHD were not observed (Table 3).

**Table 3:** Type, Source & Perceived Basis of IHD by Gender

	Male n = 44 (%)		Female n = 60 (%)		p values*
**Type of IHD**					
Inappropriate verbal comments	40	(90.9)	58	(96.7)	0.24
Work as punishment	17	(38.6)	12	(20.0)	0.047
Privileges/opportunities taken away	3	(6.8)	16	(26.7)	0.01
Recrimination for reporting	3	(6.8)	4	(6.7)	1.00
Inappropriate/unwanted physical contact	2	(4.5)	3	(5.0)	1.00
Sexual harassment	2	(4.5)	1	(1.7)	1.00
**Source of IHD**					
Specialist Physicians	33	(75.0)	47	(78.3)	0.84
Hospital Nurses	21	(47.7)	36	(60.0)	0.24
Specialty Residents	16	(36.4)	31	(57.7)	0.16
Patients	16	(36.4)	20	(33.3)	0.84
Family Physicians	7	(15.9)	12	(20.0)	0.80
Support Staff	4	(9.1)	10	(16.7)	0.38
Family Medicine Residents	3	(6.8)	4	(6.7)	1.00
Program Director	2	(4.5)	2	(3.3)	1.00
Family Medicine Nurses	1	(2.3)	1	(1.7)	1.00
**Perceived Basis for IHD**					
Gender	2	(4.5)	25	(41.7)	0.00001
Ethnicity	4	(9.1)	13	(21.7)	0.11
Culture	4	(9.1)	6	(10.0)	1.00
Language	1	(2.3)	4	(6.7)	0.39
Sexual Orientation	1	(2.3)	0	(0.0)	0.42

*Fisher's Exact test

Note: Respondents could check as many responses as apply, thus response totals add up to more than the column totals noted at the top.

### Age

An ANOVA using age as the dependent variable and IHD (Yes = 103; No = 127) as the independent variable revealed that those reporting IHD were younger (Mean age = 33.7; SD = 4.8) than those not reporting IHD (Mean age = 35.2; SD = 5.7), p = 0.034. The relationship between age and IHD was explored further using logistic regression. Using the age categories defined in Table 1 the odds of reporting or experiencing IHD was 0.76 (CI = 0.59 - 0.98), p = 0.036, with each unit increment in age category.

### Canadian and International Medical Graduates

There was no significant difference in the proportion of Canadian (46.1%, 88/191) and IMGs (41.0%, 16/39) who experienced IHD. While the type and source of IHD were similar between the two groups, significant differences were noted in the perceived basis of the IHD (Table 4). A significantly greater proportion of IMGs perceived ethnicity, culture, or language to be the basis of IHD. There was a trend for Canadian family medicine graduates to identify gender as a basis for IHD, but this variable did not reach statistical significance (p = 0.06).

**Table 4:** IHD by Canadian vs International Medical Graduates

	Canadian n = 88 (%)		IMG n = 16 (%)		p values*
**Type of IHD**					
Inappropriate verbal comments	82	(93.2)	16	(100.0)	0.59
Work as punishment	27	(30.7)	2	(12.5)	0.22
Privileges/opportunities taken away	15	(17.0)	4	(25.0)	0.49
Recrimination for reporting	5	(5.7)	2	(12.5)	0.29
Inappropriate/unwanted physical contact	5	(5.7)	0	(0.0)	1.00
Sexual harassment	3	(3.4)	0	(0.0)	1.00
**Source of IHD**					
Specialist Physicians	70	(79.5)	11	(68.8)	0.34
Hospital Nurses	50	(56.8)	7	(43.8)	0.42
Specialty Residents	39	(44.3)	9	(56.3)	0.42
Patients	34	(38.6)	2	(12.5)	0.05
Family Physicians	14	(15.9)	5	(31.3)	0.16
Support Staff	13	(14.8)	1	(6.3)	0.69
Family Medicine Residents	5	(5.7)	2	(12.5)	0.29
Program Director	4	(4.5)	0	(0.0)	1.00
Family Medicine Nurses	2	(2.3)	0	(0.0)	1.00
**Perceived Basis for IHD**					
Gender	27	(30.7)	1	(6.2)	0.06
Ethnicity	8	(9.1)	9	(56.3)	0.00006
Culture	4	(4.5)	6	(37.5)	0.0008
Language	1	(1.1)	4	(25.0)	0.002
Sexual Orientation	1	(1.1)	0	(0.0)	1.00
					

*Fisher's Exact test

Note: Respondents could check as many responses as apply, thus response totals add up to more than the column totals noted at the top.

## Discussion

This study exploring IHD within a Canadian family medicine residency program setting reveals that perceptions of IHD are prevalent among family medicine graduates. Although the overall prevalence of IHD in our study (44.7%) is at the lower end of that reported in the literature [1,11,20,35], clearly any occurrence of IHD is of concern. Our finding that inappropriate verbal comments were the most frequent type of IHD is consistent with the published literature [11]. The study also provides new information on the comparison of IHD between Canadian and IMG family medicine graduates. While the two groups demonstrate a similar prevalence of IHD, a greater proportion of IMGs perceive ethnicity, culture, or language to be the basis of IHD and Canadian graduates tend to identify gender as the basis of IHD.

The primary sources of IHD during family medicine residency training appear to be external to the discipline of family medicine: specialist physicians, hospital nurses and specialty residents. This finding is consistent with another study that reported abuse during clerkship rotations to be most prevalent on surgical rotations [36]. Within the hospital setting, there is a traditional power structure and hierarchy in which family medicine residents may be at the lower end of the resident pecking order and, in a values-challenged system, possibly less deserving of respect [6,10,13]. Predominant perpetrators of mistreatment in medical education settings have been reported to be consultants, supervisors, instructors, physicians, colleagues, nurses, allied health personnel, and sometimes patients [6,20,37]. Some may use teaching as a forum to humiliate, rather than to constructively challenge and critique [8,22,26].

The finding that there are perpetrators from within the discipline of family medicine is also of great concern, particularly when these individuals are entrusted with teaching residents and influence local family medicine culture or practice. There is substantial room and need for constructive dialogue and conjoint behaviourally-based problem solving on matters of IHD, both within and external to family medicine.

Gender and ethnicity were cited as the most frequent perceived basis for IHD, with more females citing gender and more IMGs citing ethnicity, culture, and language. The gender differences may arise from biological or cultural differences in the perception, specific identification, and recollection of an IHD event. In some medical environments, there may be a perceived or real "boys club" mentality wherein female residents need to "prove" themselves. Some studies reveal significant differences in the degree of sexual discrimination and sexual harassment across specialties in American medical schools [6,38]. Power differentials may exist between female residents and male physicians or experienced nurses. Females may perceive that the consequences of reporting IHD is worse than the IHD itself, for the rewards of being labelled a "troublemaker" are few. We propose that "work as punishment" (i.e. being given additional work duties), experienced by more males than females, is a more aggressive type of IHD compared to the passive-aggressive removal of privileges (i.e. being subtly excluded from procedural skill learning opportunities) experienced by more females.

Given the many challenges that IMGs experience in workforce integration in Canada, it is reassuring that no significant difference exists in the proportion of Canadian and IMG graduates who experienced IHD in our setting. We speculate that, at the time of the survey, perhaps significant Albertan and pan-Canadian IMG focused efforts were beginning to bring about change at the point of learning and care [39]. The greater proportion of IMGs perceiving ethnicity, culture or language to be the basis of IHD is not surprising, although disturbing given both Canada's reputation as a tolerant and multi-cultural society, the Canadian Charter of Rights and Freedoms, and the societal thrust towards equity in education. Family medicine residency programs need to be cognizant of IHD experiences by IMGs and proactively formulate a constructive and robust educational response.

Our study findings reveal that that older graduates are less likely to report IHD than their younger colleagues. It is unknown whether older graduates truly experience less unwanted behaviours or whether with increasing age and maturity, perceptions and/or interpretations change. Behaviour that may be perceived as a form of IHD by a younger graduate may be "taken in stride," discounted or ignored by one who is older. It is also possible that perpetrators of IHD may be more likely to target younger individuals. Further research examining the relationship between age and IHD using qualitative methods may help to elucidate these findings.

Our finding that only slightly more than half (54.4%) of family medicine graduates knew about the process to address IHD in residency training is not reassuring. This may partially explain why official grievance avenues are under utilized [21]. In both family medicine training programs and in the postgraduate medical education and university systems in which residency training is embedded, there are policies and programs to foster an educational environment that is both conducive to learning and explicitly supportive of the well-being of learners. Consequently, it is prudent to communicate within training programs that prevention [21] is the preferred strategy. Efforts by residency programs to monitor resident well-being are recommended as well-being may be a good indicator of progress in eradicating IHD.

The impact of IHD on the graduates in this study is unknown. Consequently, future research on how family medicine residents cope with IHD would be beneficial especially in understanding what type of support mechanisms should be provided. Evidence suggests that male students who are abused are more likely to perpetuate mistreatment, even toward patients [40]. Whether residents in our study who were intimidated or harassed went on to intimidate others is unknown and may be worthy of investigation.

Is more IHD policy needed? We doubt it. Quinn [41] asserts that the connection between law, policies and everyday practice is "contradictory and incomplete". It appears that the real policy challenge is meaningful and enlightened implementation at the point of learning and care. Educators addressing IHD issues recognize the power differential and hierarchical systems that can make it difficult for a learner to directly attempt to address a personally-directed IHD. It is agonizing to challenge a superior if that same individual is the one who will be completing the end of rotation evaluation. What is difficult at the level of an individual learner must be addressed by educators, local educational structures, and those in authority. While solutions to IHD issues were not the focus of the current study, promoting both a healthy learning environment and physician wellness could be a constructive contextual approach in facilitating preventative efforts.

### Limitations

This study was limited by the retrospective nature of the survey. Recall bias is likely given that graduates were asked to remember experiences of IHD that may have occurred 5-7 years prior. The data reflects graduates' perceptions of IHD that occurred several years ago and may not accurately reflect the current situation. For this reason, ongoing examination of IHD is necessary.

The study questionnaire did not operationally define IHD nor its specific types; therefore, it is likely that respondents interpreted IHD differently. Furthermore the survey options for the primary basis of IHD may have been limiting in that many "other" responses were generated by the respondents. The questionnaire was limited to only assessing self-reported perceptions or experiences of IHD, not objectively observable or verifiable IHD events. However, this is not a major concern given the importance of opinion and perceptions in the area of IHD. It is possible that some graduates may have used the questions on IHD to report grievance or discontent with the program retrospectively, which would lead to an overestimation of the actual occurrence of IHD. The converse may have also occurred, with painful past experiences being minimized or forgotten. The IMG sample is relatively small, thus the reliability and generalisability of the findings on IHD experienced by IMGs should be interpreted with caution.

## Conclusions

Perceptions of IHD are prevalent among graduates who have completed family medicine residency training in Alberta. The most frequent type of IHD experienced was in the form of inappropriate verbal comments. The primary sources of IHD during family medicine residency training appear to be external to the discipline of family medicine. This study provides new information on the comparison of IHD between Canadian and IMG family medicine graduates, with a greater proportion of IMGs perceiving ethnicity, culture, or language to be the basis of IHD. Residency programs and the educational systems in which they are embedded should both explicitly recognize and robustly address any IHD concerns while actively promoting prevention, with an emphasis on personal and professional well being and a supportive learning environment.

## Competing interests

We declare we have no competing interests.

## Authors' contributions

RAC was the principal investigator and was involved in all aspects of the study including study design, questionnaire development, interpretation of the study findings, and manuscript preparation. OS contributed to the development of the study design, questionnaire development, ethics application, interpretation of study findings, and manuscript preparation. WW participated in the study design, questionnaire development, conducted the data analysis and contributed to manuscript preparation. FC conducted the literature review and provided assistance during the implementation phase and contributed to manuscript preparation. CH provided project management expertise in all phases of the study and contributed to data interpretation and manuscript preparation. All the authors contributed to critical revisions and approved the final version of the manuscript.

## Pre-publication history

The pre-publication history for this paper can be accessed here:

http://www.biomedcentral.com/1472-6920/11/88/prepub
